# Molecular co-catalyst accelerating hole transfer for enhanced photocatalytic H_2_ evolution

**DOI:** 10.1038/ncomms9647

**Published:** 2015-10-21

**Authors:** Wentuan Bi, Xiaogang Li, Lei Zhang, Tao Jin, Lidong Zhang, Qun Zhang, Yi Luo, Changzheng Wu, Yi Xie

**Affiliations:** 1 Hefei National Laboratory for Physical Sciences at the Microscale, Collaborative Innovation Center of Chemistry for Energy Materials, Synergetic Innovation Center of Quantum Information and Quantum Physics, Hefei Science Center of CAS, CAS Key Laboratory of Mechanical Behavior and Design of Materials, University of Science and Technology of China, Hefei 230026, Anhui, China; 2 National Synchrotron Radiation Laboratory, University of Science and Technology of China, Hefei 230026, Anhui, China

## Abstract

In artificial photocatalysis, sluggish kinetics of hole transfer and the resulting high-charge recombination rate have been the Achilles' heel of photocatalytic conversion efficiency. Here we demonstrate water-soluble molecules as co-catalysts to accelerate hole transfer for improved photocatalytic H_2_ evolution activity. Trifluoroacetic acid (TFA), by virtue of its reversible redox couple TFA·/TFA^−^, serves as a homogeneous co-catalyst that not only maximizes the contact areas between co-catalysts and reactants but also greatly promotes hole transfer. Thus K_4_Nb_6_O_17_ nanosheet catalysts achieve drastically increased photocatalytic H_2_ production rate in the presence of TFA, up to 32 times with respect to the blank experiment. The molecular co-catalyst represents a new, simple and highly effective approach to suppress recombination of photogenerated charges, and has provided fertile new ground for creating high-efficiency photosynthesis systems, avoiding use of noble-metal co-catalysts.

## Abstract (web summary)

Enhancing the kinetics of hole transfer at photocatalytic surfaces serves to promote the overall efficiency closer to practically implementable levels. Here, the authors employ trifluoroacetic acid to achieve this goal and significantly improve the photocatalytic H_2_ evolution activity of K_4_Nb_6_O_17_.

## Graphical abstract

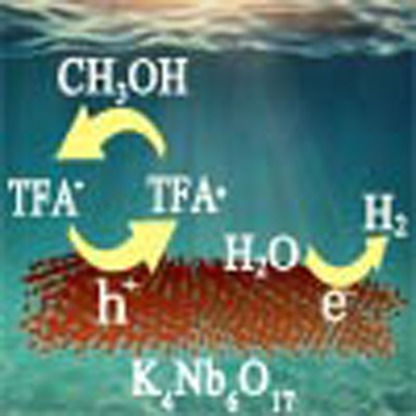

Photocatalytic conversion of solar energy to fuels is considered to be an ideal, renewable energy resource for the future, yet the relatively low-energy conversion efficiency remains the most critical factor that limits its practical applications^1^,^2^,^3^,^4^. In principle, the generation of electron–hole pairs in photocatalysts upon illumination and their participation in subsequent redox reactions are the main processes among the widely investigated photocatalytic reactions. To achieve highly efficient photochemical conversion, the main challenge in photocatalysis is to maximize the extraction of charge carriers while suppressing their recombination^5^,^6^. Arguably, the electron transfer is typically sufficiently fast, whereas the transport of hole and subsequent reactions are generally slow, eventually leading to the high-charge recombination rate^7^,^8^,^9^. From this perspective, accelerating hole transfer kinetics, typically the rate-limiting step, has emerged as the inevitable route to achieve high-efficiency charge separation.

Co-catalysts are known to provide trapping sites for the photogenerated charges and promote charge separation^10^,^11^. Noble-metal oxides (such as RuO_2_ and IrO_2_) are usually used as co-catalysts for hole transport because they can effectively lower the overpotential for the oxidation reaction. Meanwhile, subjected to their high costs of upscaling, several cost-effective and earth-abundant alternatives (primarily based on Co-Pi, NiO_*x*_, CoO_*x*_, and so on) have also been exploited and achieve relatively high efficiency. However, this strategy through solid-state co-catalysts loading is potentially limited by finite contact areas between co-catalysts and reactants, lacking sufficient active sites for catalysis.

Alternatively, homogeneous catalysis, free from limited contact areas, has gained considerable attention due to its high activity and selectivity^12^. For example, biological hydrogenases and their artificial mimics as well as the newly developed transition-metal complexes are regarded as a class of novel H_2_-evolution catalysts^13^,^14^. The inspiration gained from Mn_4_CaO_5_ clusters of photosystem II in natural photosynthesis also stimulates the exploitation of O_2_-evolution catalysts^15^. Catalysts of this type have demonstrated impressive achievements in artificial photocatalysis. Nevertheless, note that most of these photocatalytic processes must be carried out in organic systems due to the poor water solubility and low stability of organometallic molecules, which is neither economical nor environmentally benign. To this end, developing water-soluble co-catalysts in photocatalytic systems is indispensable to achieve cost-effective and highly efficient artificial photocatalysis.

In this article, we propose a water-soluble molecular co-catalyst strategy to accelerate the hole transfer kinetics. The reversible redox couple TFA·/TFA^–^, making trifluoroacetic acid molecule (TFA) a robust molecular co-catalyst, provides further charge separation pathway across the catalysts interface, and consequently facilitates photocatalytic H_2_ generation. *In situ* electron spin resonance (ESR) together with energy-level alignment reveal that photogenerated holes in K_4_Nb_6_O_17_ valence band readily react with the adsorbed TFA anions to yield TFA radicals and simultaneously the highly reactive radicals transfer holes to methanol, eliminating the main competitive pathway of electron–hole recombination. Moreover, ultrafast transient absorption spectroscopy combined with static as well as time-resolved photoluminescence spectroscopy confirms that effective charge separation is the primary factor behind the dramatically improved photocatalytic performance. Homogeneous co-catalyst, which is free from the limited contact areas between co-catalysts and reactants, provides sufficient active sites for catalysis, thus offering new opportunity to develop high-efficiency photosynthesis.

## Results

### K_4_Nb_6_O_17_ nanosheets

Layered niobate is a well-accepted photocatalytic system for water splitting, and its unique layered structure has boosted new opportunities for the manipulation of two-dimensional nanomaterials by intercalation and delamination^4^,^16^,^17^,^18^. Benefiting from the highly anisotropic structure, niobate ultrathin nanosheets allow advances in potentially exposing more reactive sites and facilitating the contact of catalysts with reactants, and thus provide an ideal platform for artificial photocatalysis. Here K_4_Nb_6_O_17_ nanosheet catalysts were synthesized via a conventional solution method by anisotropic growth^19^. The as-synthesized K_4_Nb_6_O_17_ catalysts showed a typical nanosheet morphology with lateral size varied from 100 to 500 nm, as shown by scanning electron microscopy (SEM) and transmission electron microscopy (TEM; Supplementary Fig. 1). In addition, the well-resolved lattice features in high-resolution transmission electron microscope (HRTEM) image revealed the high crystallinity of these nanosheets and the interplanar distances coincided well with that of (200) and (002) facets respectively.

### Photocatalytic performance

**Figure 1:**
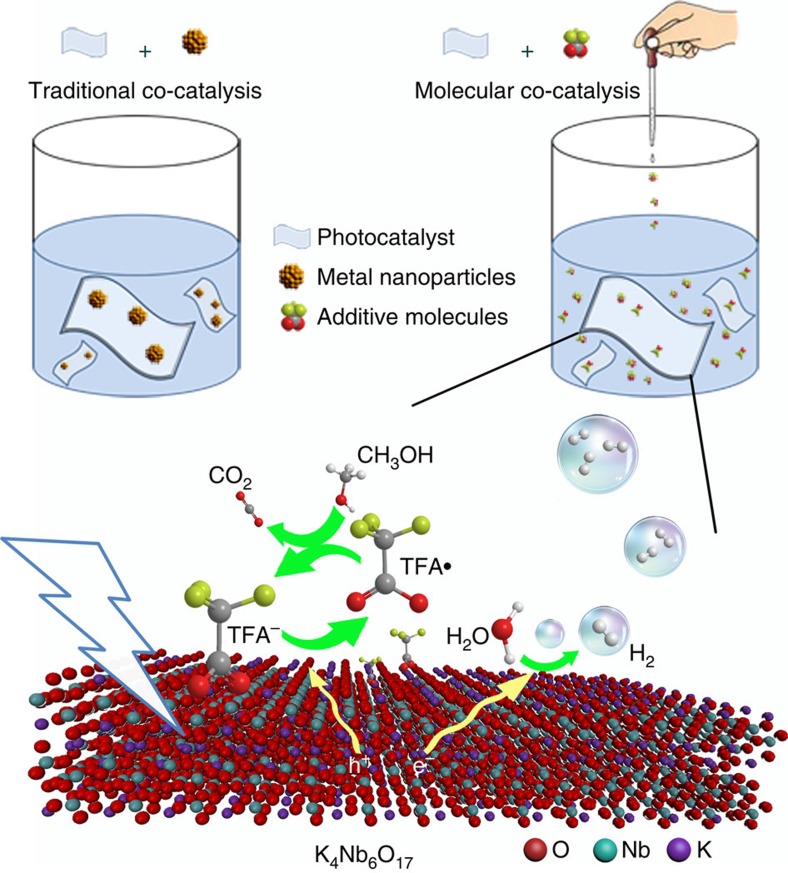
Schematic illustration of molecular co-catalyst strategy for accelerating hole transfer. Homogeneous molecular co-catalysts use reversible redox couple and highly active free radical reactions to promote hole transport, unlike supported heterogeneous co-catalysts, which is constrained by finite contact areas between co-catalysts and reactants. Purple, cyan and red spheres denote K, Nb and O atoms, respectively.

**Figure 2:**
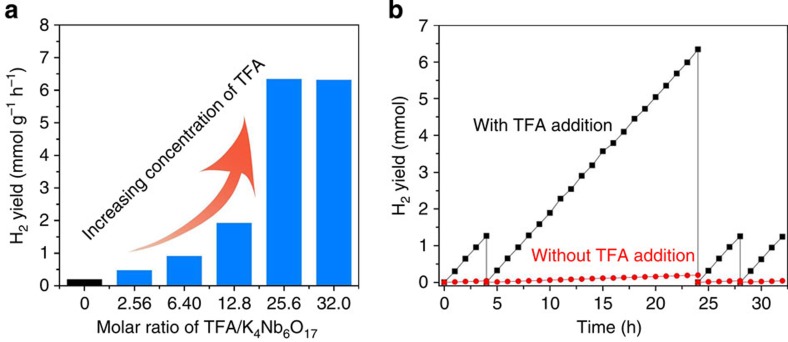
Photocatalytic H_2_ production performance. (**a**) Photocatalytic activity on K_4_Nb_6_O_17_ nanosheet catalysts with different molar ratio of TFA/K_4_Nb_6_O_17_. (**b**) Cycle stability test on K_4_Nb_6_O_17_ nanosheet catalysts with 100 μl TFA addition. Reaction condition: 50 mg catalyst, 200 ml 20 vol% methanol aqueous solution and a specific amount of TFA, under irradiation of a 300 W Xe lamp.

As illustrated in Fig. 1, the reversible redox couple TFA·/TFA^–^ and the highly active intermolecular radical reactions make TFA molecule a robust hole shuttle, effectively promoting the charge separation of K_4_Nb_6_O_17_ catalysts, and thus leading to improved photocatalytic activity. Figure 2a shows the comparison of H_2_ evolution rates on K_4_Nb_6_O_17_ nanosheet catalysts with extra addition of different amounts of TFA as molecular co-catalyst. K_4_Nb_6_O_17_ nanosheets alone showed low activity in photocatalytic H_2_ evolution (ca.195 μmol g^−1^ h^−1^). Notably, when TFA as co-catalyst was added, the H_2_ evolution activity turned out to be dramatically enhanced. Substantial increase of H_2_ evolution rates was observed with the molar ratio of TFA/K_4_Nb_6_O_17_ ranging from 2.56 to 25.6, reaching a maximum value of 6,344 μmol g^−1^ h^−1^, up to 32 times with respect to that of bare K_4_Nb_6_O_17_ nanosheets without TFA. Notably, increasing the molar ratio of TFA/K_4_Nb_6_O_17_ above 25.6 did not bring about further obvious increase for photocatalytic performance which is probably due to the saturated adsorption of TFA molecules on the surface of K_4_Nb_6_O_17_ catalysts, as confirmed by elemental analysis in Supplementary Table 1 and Supplementary Note 1. In a word, niobate photocatalyst achieved a significantly improved H_2_ generation rate when water-soluble TFA molecule was used as co-catalyst.

Recycling test of photocatalytic H_2_ evolution on the K_4_Nb_6_O_17_ nanosheet catalysts with addition of 100 μl TFA was performed to evaluate the photocatalytic stability during a long-term photocatalytic reaction. As shown in Fig. 2b, the respective total amount of H_2_ after 20 h illumination was 6.344 mmol and no noticeable degradation was detected during the four consecutive reactions, which indicates the high stability of K_4_Nb_6_O_17_ catalysts and that no obvious side reactions associated with TFA consumption occurred during the photocatalytic process. The reversibility of TFA was further verified by ^19^F NMR. As illustrated in Supplementary Fig. 2 and Supplementary Note 2, no new fluoride species were detected after the reaction. And according to the quantitative characterization results, the concentration of TFA remains constant during the whole photocatalytic process.

To disclose the role of TFA molecules in the photocatalytic H_2_ evolution reaction, a series of control experiments were carried out for comparison. Control experiment without methanol but with only 100 μl TFA (Supplementary Fig. 3a) showed a relatively low activity in photocatalytic H_2_ evolution (ca. 158 μmol g^−1^ h^−1^), indicating a synergistic effect exists between TFA molecules and CH_3_OH. Meanwhile, to rule out the influence from the changes of hydrogen ion concentration induced by TFA addition, we systematically investigated the pH effect by adjusting pH values of the solution with 1 M HCl. As shown in Supplementary Fig. 3b, the H_2_ evolution rates were found to slightly increase with decreasing the pH values, but well below 600 μmol g^−1^ h^−1^. These results indicate that TFA indeed directly participated in the photocatalytic reaction process, rather than determining the pH value of the catalytic system only. Based on the above control experiments, it is found that the added TFA should be responsible for the significant performance improvement of photocatalytic system, in which TFA molecules recycle stoichiometrically during the reaction process.

### K_4_Nb_6_O_17_ nanosheets upon TFA addition

To gain in-depth insight into the origin of the improved photocatalytic activity, we compared the morphology, crystalline structure, surface chemical states and optical properties of the K_4_Nb_6_O_17_ nanosheets with/without addition of TFA after the catalytic reaction. SEM and TEM images in Supplementary Fig. 1 revealed that K_4_Nb_6_O_17_ catalysts still kept the initial nanosheet morphology after reaction. X-ray powder diffraction and HRTEM further confirmed structural maintenance. High-resolution X-ray photoelectron spectroscopy analysis of the K_4_Nb_6_O_17_ nanosheet catalysts after TFA addition (Supplementary Fig. 4a) showed an additional peak at 688.6 eV which can be attributed to the -CF_3_ groups, indicating the preferential adsorption of TFA on the surface of K_4_Nb_6_O_17_ nanosheets^20^. The optical properties were measured using ultraviolet–visible absorption spectra in the wavelength range of 220–800 nm (Supplementary Fig. 4b). The absorption edge of K_4_Nb_6_O_17_ nanosheet catalysts was observed at ∼348 nm, corresponding to a bandgap of 3.56 eV. Upon TFA addition, a weak absorption enhancement in the ultraviolet region was observed, which probably originates from the absorption of TFA itself, whereas the absorption edge of photocatalysts showed no noticeable shift. This result demonstrates that the improved photocatalytic performance cannot be ascribed to the enhancement of light absorption, but instead to the effective charge separation.

### TA and PL analyses

**Figure 3:**
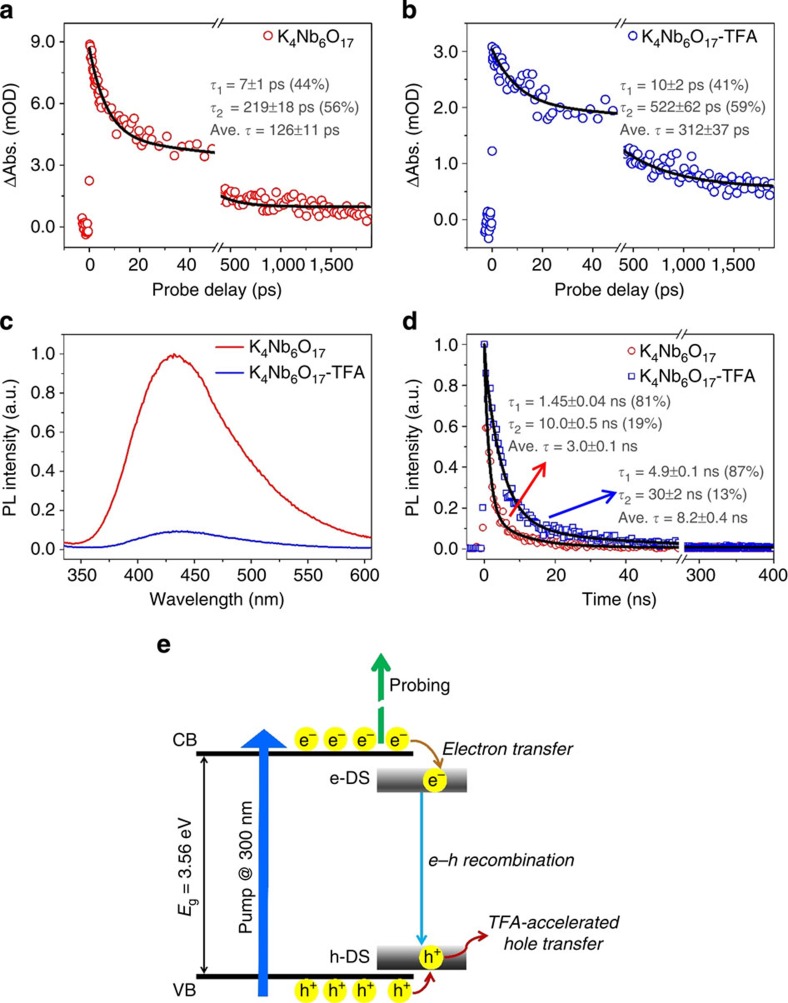
Spectroscopic evidence for effective charge separation process. Representative ultrafast TA kinetics probed at 500 nm (pump at 300 nm) for K_4_Nb_6_O_17_ nanosheets in the (**a**) absence and (**b**) presence of TFA. The TA signal (that is, the absorbance changes, or ΔAbs. in short) is given in mOD where OD stands for optical density. (**c**) PL emission and (**d**) time-resolved PL spectra (excitation at 315 nm, emission at 430 nm) for both K_4_Nb_6_O_17_ and K_4_Nb_6_O_17_−TFA. (**e**) Schematic illustration of the mechanisms involved; see text for details.

To evaluate how the addition of TFA affects the charge separation behaviour involved in the K_4_Nb_6_O_17_ nanosheet catalysts, we interrogated the K_4_Nb_6_O_17_ and K_4_Nb_6_O_17_–TFA samples by means of ultrafast transient absorption (TA) spectroscopy, which is known as a robust tool for tracking in real-time charge carrier dynamics in nanosystems^21^. In our TA measurements, a femtosecond ultraviolet pump/white-light continuum probe scheme was employed (see Supplementary Methods for details of the pump–probe experiments). The centre wavelength of the pump pulses was chosen at 300 nm, which can effectively photo-induce an interband transition in the semiconductor K_4_Nb_6_O_17_ system (refer to Supplementary Fig. 4b). Since the 390−610 nm white-light continuum probe was found to yield essentially the same TA kinetics for each sample, we show here a set of representative data taken at 500 nm (Fig. 3a,b). It turned out that both samples exhibited TA signals due to photoinduced absorption, whose relaxation can be characterized by two time constants: *τ*_1_=7±1 ps and *τ*_2_=219±18 ps for K_4_Nb_6_O_17_ while *τ*_1_=10±2 ps and *τ*_2_=522±62 ps for K_4_Nb_6_O_17_–TFA (all with a roughly 40:60 percentage for the two exponential components). Note that the long-time decay component(s) cannot be identified due to the probe-delay limit of our pump–probe spectrometer (ca. 2 ns). The observed two components in the picosecond domain may reflect the electron dynamics associated with the defect states that are energetically located within the bandgap. Considering that such defect states could be long-lived (in the nanosecond domain), we resorted to photoluminescence (PL) spectroscopy. It is clearly seen from the PL spectra excited at 315 nm (Fig. 3c) that the addition of TFA results in substantial PL quenching, indicating greatly suppressed radiative electron–hole recombination in K_4_Nb_6_O_17_–TFA with respect to K_4_Nb_6_O_17_. Figure 3d compares the PL lifetimes recorded for the two samples at the emission wavelength of 430 nm (that is, the PL intensity maximum in Fig. 3c). Notably, two lifetimes with sizable components, as in the TA kinetics measurements, were observed for both samples: *τ*_1_=1.45±0.04 ns (81%) and *τ*_2_=10.0±0.5 ns (19%) for K_4_Nb_6_O_17_ while *τ*_1_=4.9±0.1 ns (87%) and *τ*_2_=30±2 ns (13%) for K_4_Nb_6_O_17_–TFA.

On the basis of the combined TA and PL observations, we illustrate in Fig. 3e the schematic mechanisms underlying the photoexcited carrier dynamics involved in the system. The observed PL emissions peaking at ca. 430 nm (that is, 2.88 eV, red-shifted with respect to the 3.56-eV bandgap) with two PL lifetimes suggest that the radiative electron–hole recombination responsible for PL could originate from two interfacial defect states (probably with different trap depths) near the conduction band (CB) bottom (labelled e-DS as a whole in Fig. 3e) that act as the PL emission centres^22^. These defect states receive and accumulate the photogenerated electrons transferred from the CB bottom in a bi-exponential relaxation manner, as evidenced by the observed TA kinetics which are obviously commensurate with the time-resolved PL results. On the other hand, the photogenerated holes are transferred from the valence band (VB) to the defect states near the VB top (labelled h-DS in Fig. 3e). The addition of TFA can be expected to accelerate the subsequent hole transfer from h-DS to hole scavengers in the solution (including methanol as well as TFA itself). This is understandable because the situation of h-DS being effectively vacated via the TFA anion-accelerated hole transfer will eventually decelerate the processes of electron–hole recombination and electron transfer from CB to e-DS, as verified by our observed, significantly altered carrier dynamics. It is worth stressing here that, in the presence of TFA, the average time describing the overall electron transfer from CB to e-DS is increased by a factor of ca. 2.48 (312±37 ps for K_4_Nb_6_O_17_–TFA versus 126±11 ps for K_4_Nb_6_O_17_) and meanwhile that describing the overall radiative e–h recombination by a factor of ca. 2.73 (8.2±0.4 ns for K_4_Nb_6_O_17_–TFA versus 3.0±0.1 ns for K_4_Nb_6_O_17_). Based on the self-consistent results of PL and TA observations (see insets in Fig. 3 for details), it is understandable that TFA mainly influences the hole transport process. And it can be safely inferred that the increased electron lifetimes in the system could offer more opportunities for photogenerated electrons to participate in the H^+^ reduction reaction.

### *In situ* ESR characterizations

**Figure 4:**
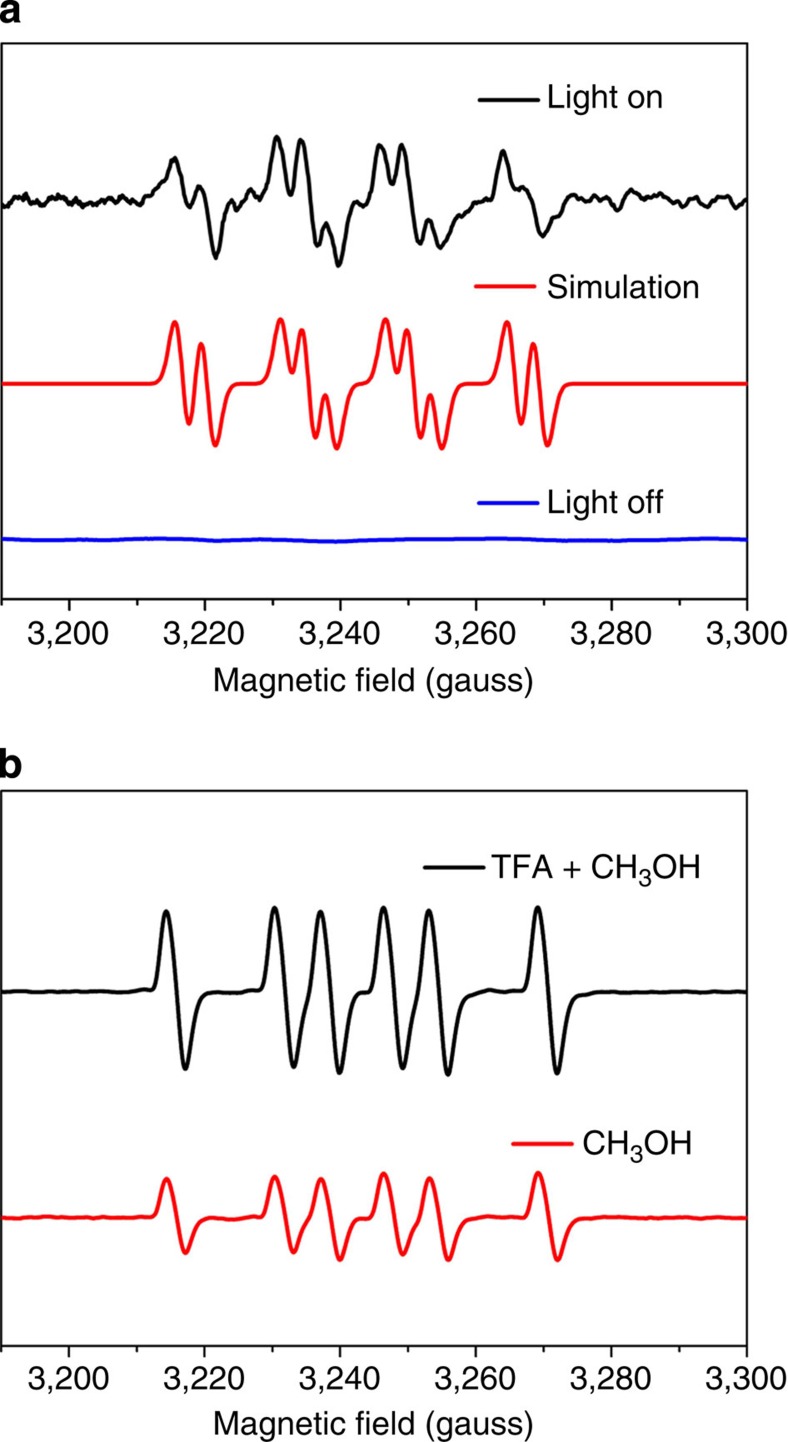
*In situ* ESR on intermediates. ESR spectra of the radicals using DMPO as the spin trapping agent (**a**) in K_4_Nb_6_O_17_ aqueous suspensions with addition of TFA; (**b**) in K_4_Nb_6_O_17_ aqueous suspensions with the presence of methanol, or methanol and TFA.

To better understand the photocatalytic process of H_2_ production in the presence of TFA, ESR technique using 5,5-dimethyl-1-pyrroline *N*-oxide (DMPO) as a trapping regent was used to *in situ* monitor the intermediates. Under ultraviolet irradiation, the typical ESR spectrum of the catalysts aqueous suspensions containing only TFA is presented in Fig. 4a, which is composed of 10 lines with hyperfine constants aN=15.5 G, aH_β_=18 G, and aF=3.6 G. These parameters exactly match the simulated ESR spectrum of DMPO–CF_3_COO adducts^23^. Control experiments in Supplementary Fig. 5 and Supplementary Note 3 also confirmed that DMPO–CF_3_COO adducts were formed via the reaction of DMPO with TFA radicals. Meanwhile, signal quenching was observed immediately when the light source was turned off, reflecting the high reactivity of DMPO–CF_3_COO adducts. Under the same illumination conditions, methanol aqueous solution with K_4_Nb_6_O_17_ nanosheet catalysts gave rise to a six-lined spectrum, which can be assigned to the DMPO–CH_3_ adducts^24^. However, as for the mixture of TFA and methanol aqueous solution, the spectrum kept the same as that in methanol aqueous solution except for a higher signal intensity, which is seen in Fig. 4b. Analogously to the reported DMPO–CH_3_ adducts formation process via the reaction of DMPO–OH adducts with dimethylsulfoxide^24^,^25^, the failure to observe the DMPO–CF_3_COO spin adducts signal may result from the ultrafast electron transfer from CH_3_OH to TFA radicals, accompanied by the generation of methyl radicals and TFA anions. Moreover, this interpretation is, in fact, substantiated by the increased intensity of DMPO–CH_3_ adducts in Fig. 4b. Based on these ESR results, it is reasonable to assert that the reversible TFA·/TFA^–^ reaction eliminates the main competitive pathway of electron–hole recombination and accelerates the oxidation of methanol, eventually promoting the hole transport process.

## Discussion

**Figure 5:**
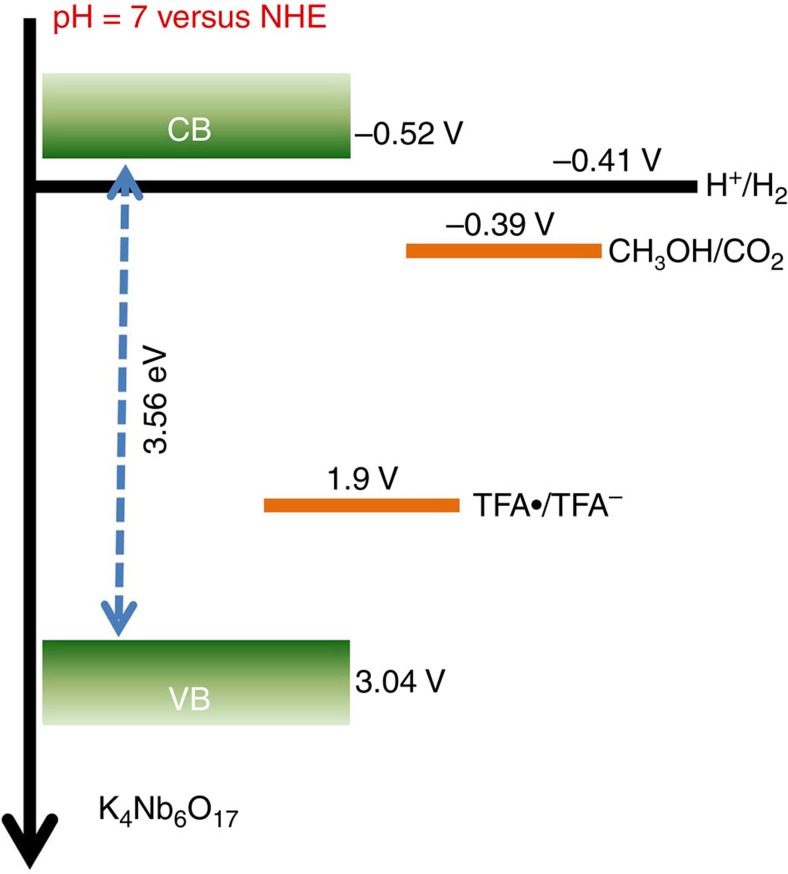
Theoretical analysis for the molecular co-catalyst strategy. Schematic illustration of the energy diagram for the K_4_Nb_6_O_17_ nanosheet catalysts and the redox potentials of TFA and CH_3_OH. NHE, normal hydrogen electrode.

Energy-level alignment illustrated in Fig. 5 provides further theoretical evidence for the above photocatalytic mechanism. According to the previous reports^26^,^27^, the flat-band potential is located just below the bottom of CB for an *n*-type semiconductor. Thus the CB potential of K_4_Nb_6_O_17_ is estimated to be −0.52 V (versus normal hydrogen electrode (NHE), pH∼7, Supplementary Fig. 6), in agreement with the previous report^17^,^28^. Given the bandgap of 3.56 eV, the VB maximum is located at 3.04 V, which is below the potential *E*(TFA·/TFA^−^)=1.90 V (Supplementary Methods) at pH=7. Therefore, the holes in K_4_Nb_6_O_17_ interfaces are able to readily react with the adsorbed TFA anions to yield TFA radicals, confirming the feasibility of TFA radicals as reactive intermediates. Meanwhile, the oxidation potential of CH_3_OH^29^, −0.39 V (versus NHE, pH∼7), is well above *E*(TFA·/TFA^–^). As such, CH_3_OH can provide electrons to reduce the highly reactive TFA radicals. In this regard, TFA molecules stand out as recyclable co-catalysts for facilitating hole transport. Given the additional charge separation pathway across the solid–liquid interface and the extremely rapid intermolecular radical reactions, charge recombination can be effectively suppressed, and therefore more electrons can be released for water reduction.

On the other hand, loading Pt co-catalysts can further optimize the photocatalytic performance by promoting proton reduction reaction. As shown in Supplementary Fig. 7, the photocatalytic H_2_ generation rate of Pt/K_4_Nb_6_O_17_ with the addition of TFA reached 9,116 μmol g^−1^ h^−1^, up to 46 times with respect to that of bare K_4_Nb_6_O_17_. Moreover, as in the aforementioned comparison of the self-consistent TA and time-resolved PL results of K_4_Nb_6_O_17_ and K_4_Nb_6_O_17_–TFA, the similar comparison of Pt/K_4_Nb_6_O_17_ and Pt/K_4_Nb_6_O_17_–TFA (Supplementary Fig. 8 and Supplementary Note 4) further confirms that TFA mainly influences the hole transfer process. Besides, it is worth mentioning that the enhanced hole transport with TFA is applicable to other metal oxide photocatalysts. Taking layered titanate Na_2_Ti_3_O_7_ as an example, photocatalytic experiments confirmed the enhanced H_2_ evolution activity with the addition of TFA, which is shown in Supplementary Figs 9,10.

**Figure 6:**
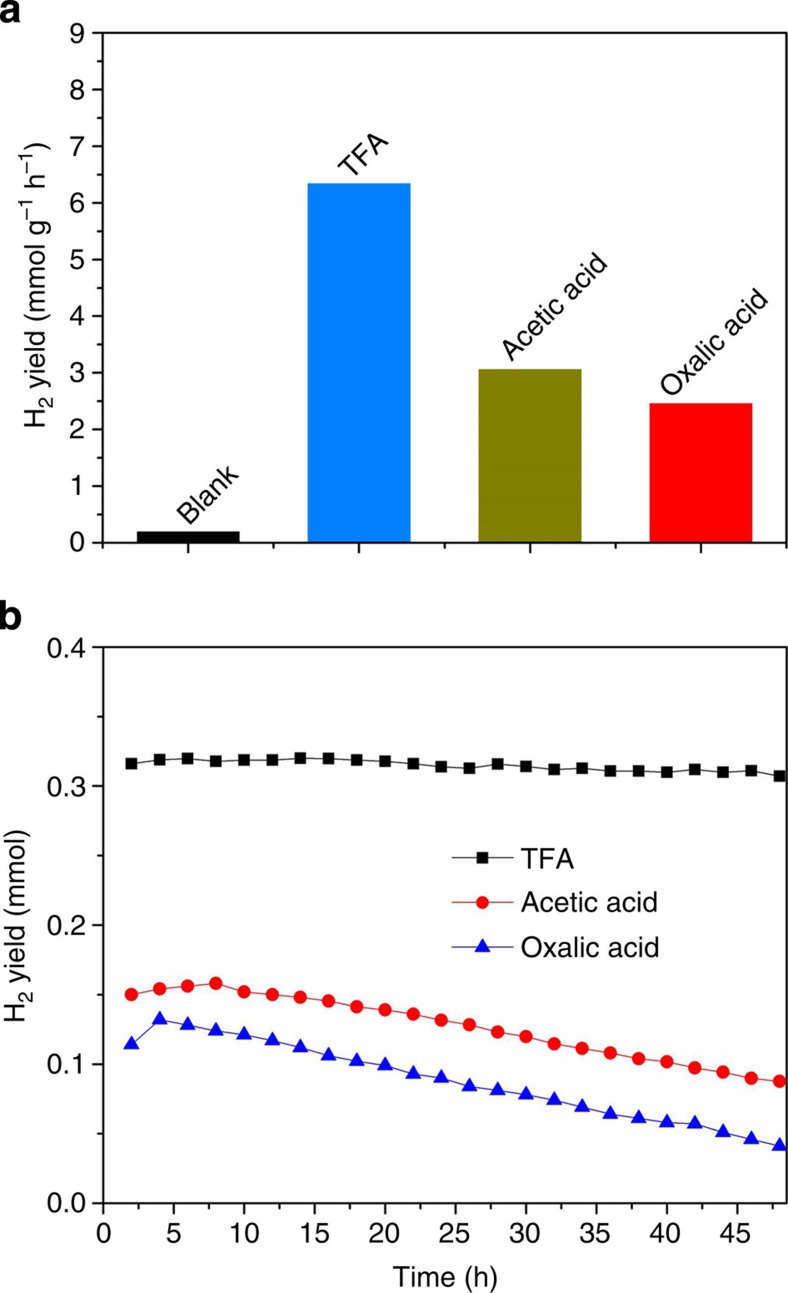
The essentials of molecular co-catalysts. Comparison of photocatalytic H_2_ evolution activity for K_4_Nb_6_O_17_ catalysts with TFA, acetic acid, oxalic acid (**a**) in the first 4 h, (**b**) every hour in the first 48 h.

To disclose the essentials of molecular co-catalysts, acetic acid and oxalic acid were also investigated. As shown in Fig. 6a, they also led to improved H_2_ production performance in the first 4 h, 15 and 12 times higher than that of bare K_4_Nb_6_O_17_ nanosheet catalysts, respectively. However, according to the ^1^H NMR in Supplementary Fig. 11 and precipitation titration results in Supplementary Note 5, both acetic acid and oxalic acid underwent irreversible oxidation, which caused obvious decrease of H_2_ generation rate in the long-time photocatalytic experiment shown in Fig. 6b. These results demonstrate that the reversibility needs to be taken into account when designing molecular co-catalysts.

In conclusion, we have proposed an efficient molecular co-catalyst strategy to accelerate hole transfer kinetics while suppressing charge recombination. Benefiting from the reversible redox couple TFA·/TFA^–^ and rapid intermolecular radical reactions, TFA molecule stands out as a homogeneous molecular co-catalyst, leading to a roughly two-fold increase of photoexcited-electron lifetime in niobate nanosheet photocatalytic system, thus eventually leading to a dramatically improved H_2_ generation rate of 6,344 μmol g^−1^ h^−1^, up to 32 times of that without the molecular co-catalyst. Altogether, the molecular co-catalyst strategy developed in this work provides a rational and cost-effective way for efficient separation of photogenerated charges, and hence paves a brand new avenue for designing high-efficiency photocatalytic reactions.

## Methods

### Synthesis of K_4_Nb_6_O_17_ catalysts

K_4_Nb_6_O_17_ nanosheet catalysts were synthesized via a conventional solution method with slight modification^19^. In a typical experiment, 0.8 g Nb_2_O_5_ powder was added into 30 ml KOH solution (1 M). After vigorous stirring for 30 min, the mixture was transferred into a 45 ml Teflon-lined autoclave, sealed and heated at 220 °C for 12–24 h. The autoclave was then allowed to cool down to room temperature naturally. The final product was washed with distilled water and ethanol alternately for several times, and then the final product was dried in vacuum at 60 °C overnight without further treatment.

### Characterization methods

The samples were characterized by X-ray powder diffraction by a Philips X'Pert Pro Super diffractometer equipped with graphite-monochromatized Cu-Kα radiation (*λ*=1.54178 Å). SEM images were performed on a Zeiss Supra 40 field-emission scanning microscope. TEM images were taken on H-7650 (Hitachi, Japan) operating at an acceleration voltage of 100 kV. HRTEM images were obtained on JEOL-2010 operating at an acceleration voltage of 200 kV. The steady-state ultraviolet–visible absorption spectra were recorded on a Perkin Elmer Lambda 950 spectrophotometer. High-resolution X-ray photoelectron spectroscopy measurements were performed on a VG ESCALAB MK II X-ray photoelectron spectrometer with an excitation source of Mg Kα=1253.6 eV. Mott–Schottky plot was measured in degassed 0.5 M Na_2_SO_4_ solution (pH=6.6) at a frequency of 10 Hz in the dark and the applied potential ranges from –0.5 to +0.5 V.

### TA spectroscopy characterizations

The ultrafast TA measurements were carried out on a modified ExciPro pump–probe spectrometer (CDP) in combination with an amplified femtosecond laser system (Coherent). All the measurements were performed under ambient conditions. The samples under investigation were the K_4_Nb_6_O_17_ film immersed in a methanol/water (1:4 vol%) mixed solution and the K_4_Nb_6_O_17_ film immersed in a methanol/water (1:4 vol%) mixed solution with a tiny amount of TFA addition (0.5 ml l^−1^). The sample cell was mounted on a rotating stage to ensure that the photoexcited volume of the sample was kept fresh during the course of the measurements. Detailed procedures for the femtosecond pump–probe experiments can be found in the Supplementary Methods.

### PL and ESR characterizations

The PL emission spectra were obtained on a FLUOROLOG-3-TAU fluorescence spectrometer (Horiba) upon excitation at 315 nm. The ns-domain time-resolved PL spectra (excited at 315 nm; monitored at the 430-nm emission) were recorded on an FLS920 fluorescence spectrometer (Edinburgh). The spin trapping experiments were performed in deoxygenated solutions using a JES-FA200 ESR spectrometer at room temperature. The irradiation experiments were carried out with a Xe lamp (500 W, USHIO Optical Modulex SX-U1501XQ).

### Photocatalytic H_2_ evolution measurements

Photocatalytic H_2_ evolution reactions were carried out in a gas-closed circulation system equipped with a vacuum line. Typically, 50 mg photocatalyst powder was dispersed in 200 ml 20 vol% CH_3_OH aqueous solution and then illuminated with a 300 W Xe lamp (PLS-SXE300/300UV, Trusttech Co., Ltd). The amount of H_2_ evolution was determined using a gas chromatography (Agilent 7890A).

## Additional information

**How to cite this article:** Bi, W. *et al*. Molecular co-catalyst accelerating hole transfer for enhanced photocatalytic H_2_ evolution. *Nat. Commun.* 6:8647 doi: 10.1038/ncomms9647 (2015).

## Supplementary Material

Supplementary Information Supplementary Figures 1-11, Supplementary Notes 1-5, Supplementary Methods and Supplementary References
