# Supplementary material for: Molecular co-catalyst accelerating hole transfer for enhanced photocatalytic H2 evolution
Source: Nat Commun. 2015 Oct 21;6:8647. doi: 10.1038/ncomms9647 (PMC4639900; doi:10.1038/ncomms9647)
Supplement: Supplementary Information — Supplementary Figures 1-11, Supplementary Notes 1-5, Supplementary Methods and Supplementary References [file ncomms9647-s1.pdf]

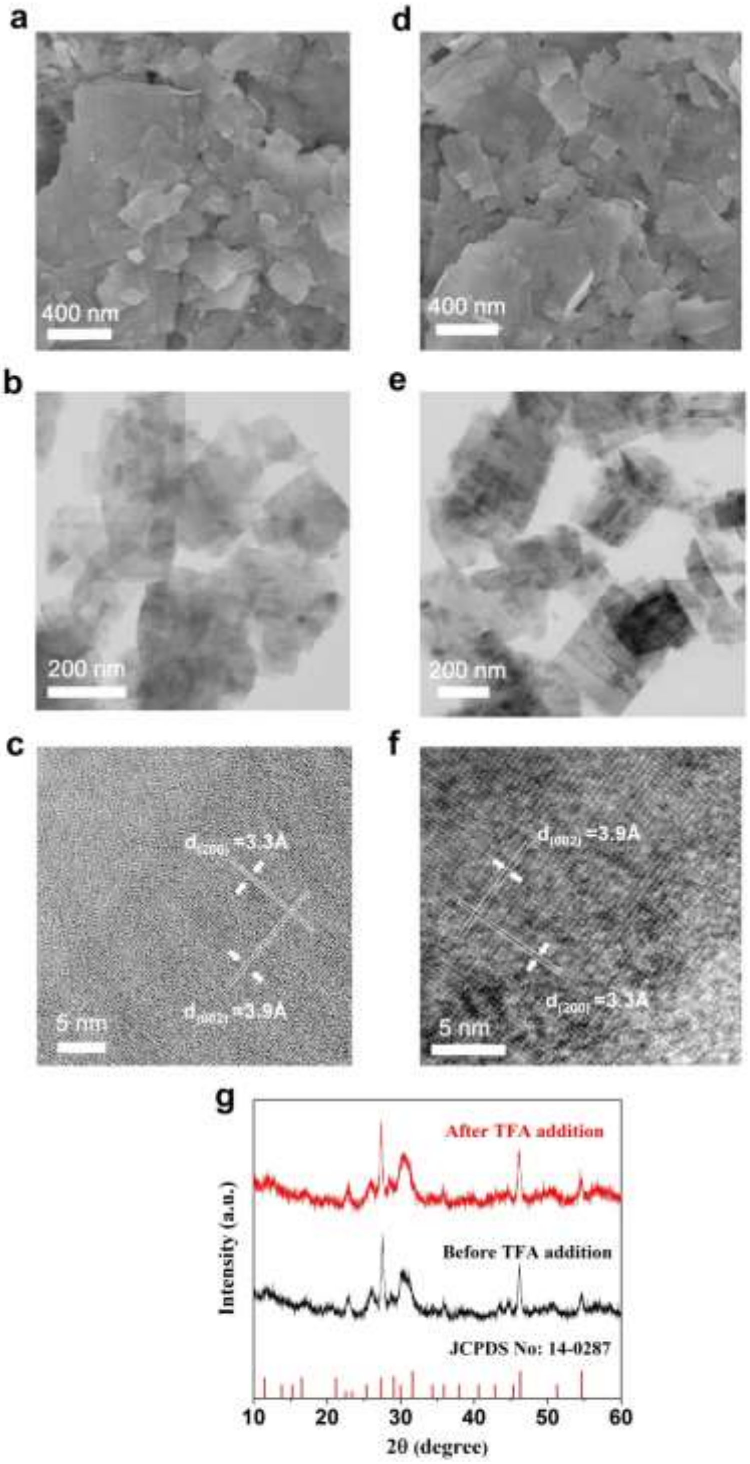

Supplementary Figure 1 | Morphological and structural comparisons of $K_4Nb_6O_{17}$ nanosheet catalysts before (a-c) and after (d-f) the addition of TFA. (a,d) SEM images, (b,e) TEM images, (c,f) HRTEM images and (g) XRD patterns.

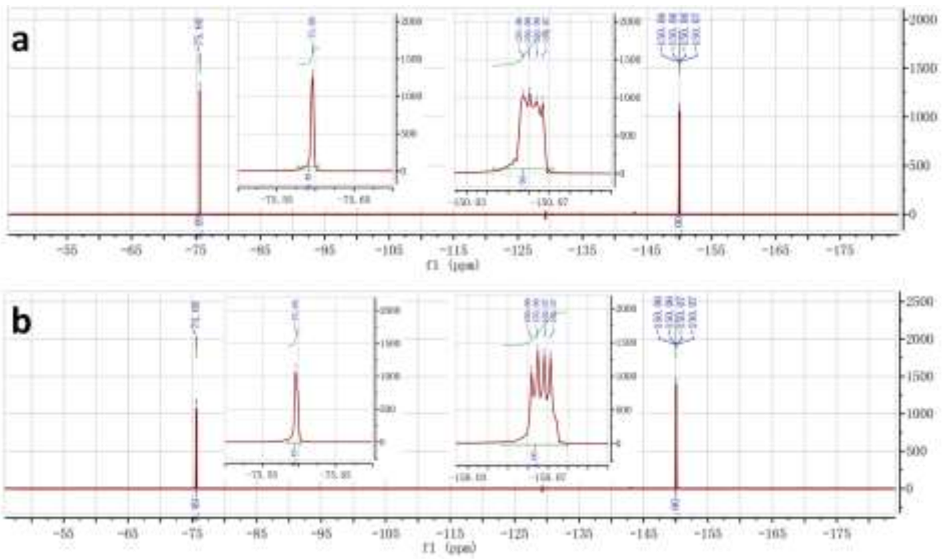

Supplementary Figure 2 | Reversibility of TFA during the photocatalytic reaction. The comparison of ^{19}F NMR spectra (a) before and (b) after 12h photocatalytic experiment. Condition: 50 mg catalyst, 100 µL TFA in 200ml 20 vol % methanol aqueous solution. Before the measurement, a known amount of NH_4BF_4 sealed in a capillary is added as an internal standard.

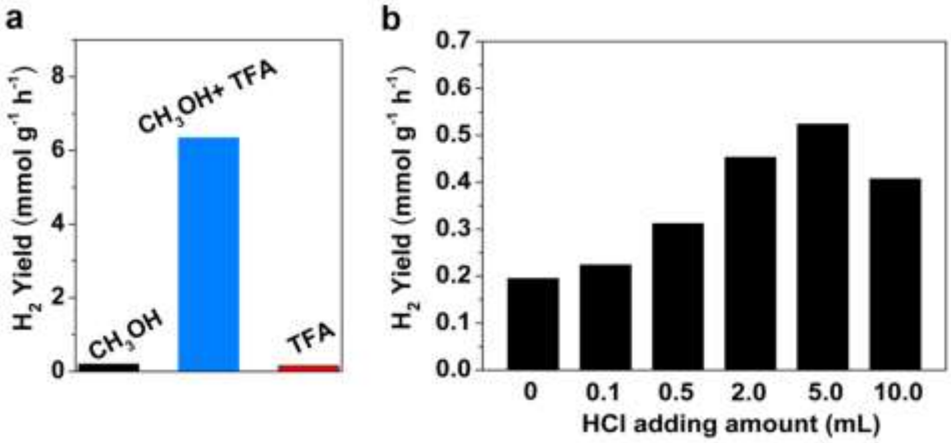

Supplementary Figure 3 | Control experiments of H_2 production activity with $K_4Nb_6O_{17}$ nanosheet catalysts. (a) Photocatalytic H_2 production activity on $K_4Nb_6O_{17}$ catalysts with 20 vol% methanol, or only 0.1 mL TFA, or mixture of 20 vol% methanol and 0.1 mL TFA. (b) H_2 production activity on $K_4Nb_6O_{17}$ catalysts with different amounts of HCl (1 M).

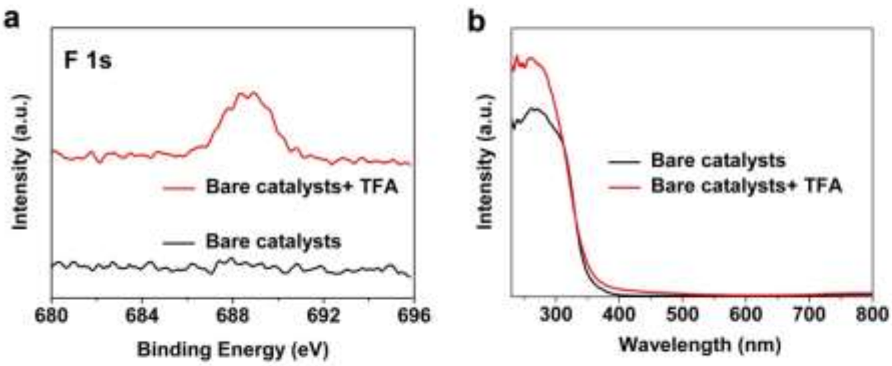

Supplementary Figure 4 | XPS and optical absorption of $K_4Nb_6O_{17}$ catalysts before and after the addition of TFA. (a) F 1s XPS spectra, (b) UV–Vis absorption spectra.

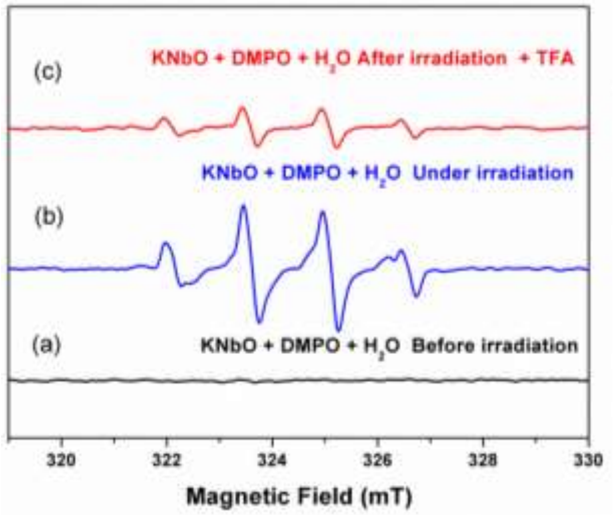

Supplementary Figure 5 | In situ ESR on radical intermediates. Experimental condition: $K_4Nb_6O_{17}$ in DMPO aqueous solution without additional hole scavenger (a) before irradiation, (b) under irradiation, (c) after irradiation and then adding TFA.

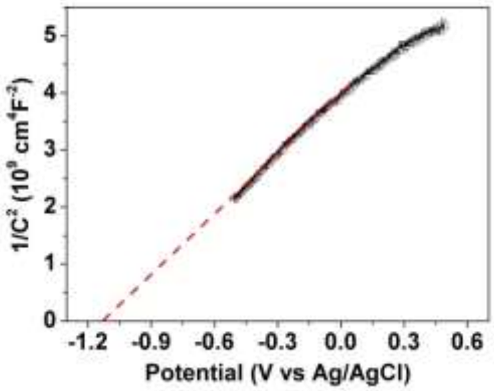

Supplementary Figure 6 | Mott–Schottky plot of $K_4Nb_6O_{17}$ catalysts. The flat band potential of $K_4Nb_6O_{17}$ determined from the Mott–Schottky plot is estimated to be −1.13V (vs. Ag/AgCl, pH~7), or −0.52 V (vs. NHE, pH~7).

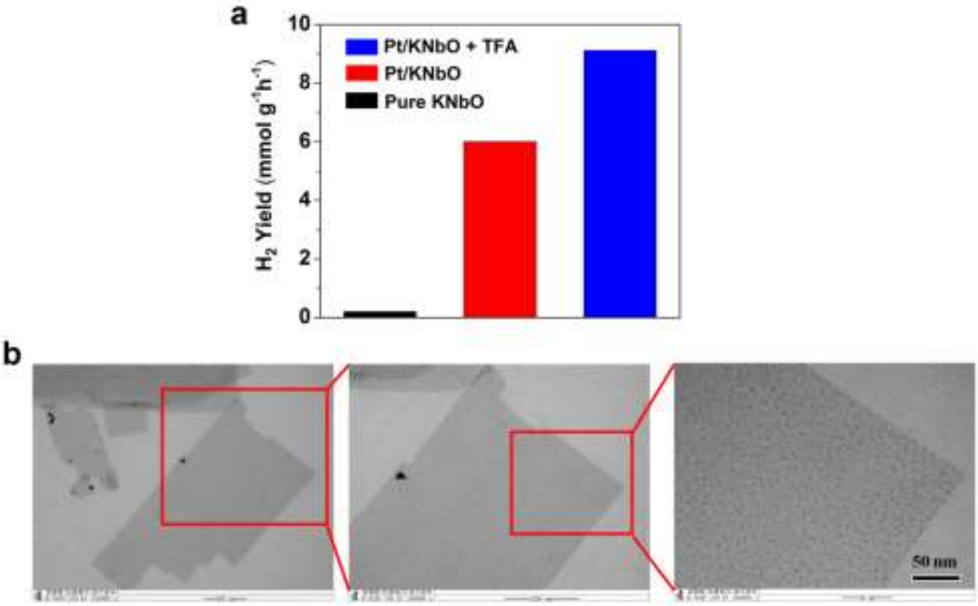

Supplementary Figure 7 | Photocatalytic performance and morphological feature of Pt/$K_4Nb_6O_{17}$. (a) The comparison of H_2 evolution activity of pure $K_4Nb_6O_{17}$, Pt/$K_4Nb_6O_{17}$, and Pt/$K_4Nb_6O_{17}$ with the addition of 100 µl TFA. (b) The typical TEM images of Pt/$K_4Nb_6O_{17}$, showing a complete surface coverage of Pt nanocrystals.

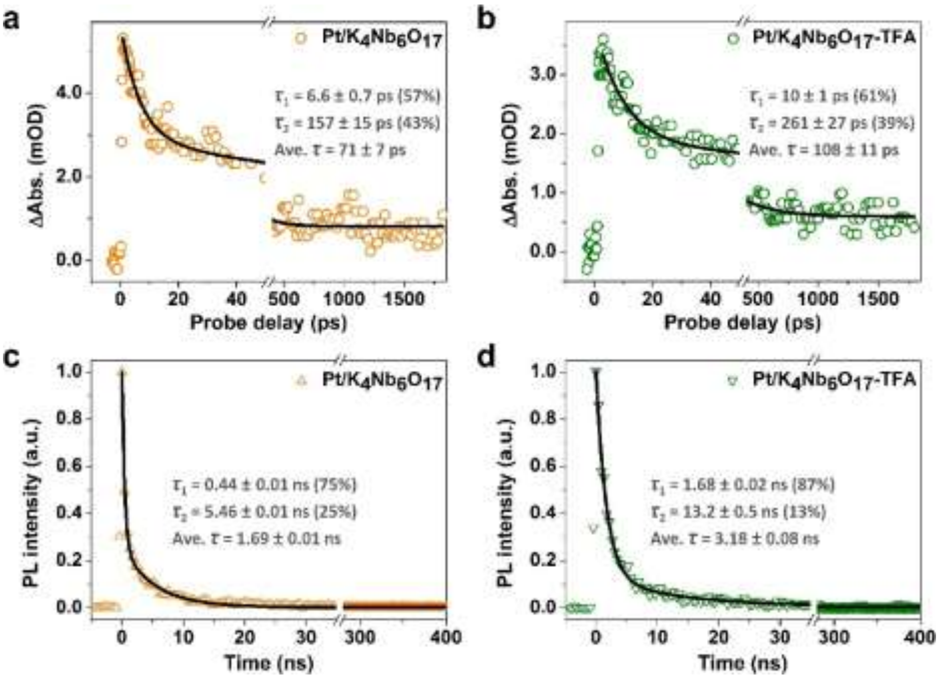

Supplementary Figure 8 | The charge carrier dynamics of Pt/$K_4Nb_6O_{17}$ nanosheets in the absence and presence of TFA. Representative ultrafast TA kinetics probed at 500 nm (pump at 300 nm) for Pt/$K_4Nb_6O_{17}$ nanosheets in the (a) absence and (b) presence of TFA. The TA signal (i.e., the absorbance changes, or ΔAbs. in short) is given in mOD where OD stands for optical density. Time-resolved PL spectra (excitation at 315 nm, emission at 430 nm) for (c) Pt/$K_4Nb_6O_{17}$ and (d) Pt/$K_4Nb_6O_{17}$-TFA.

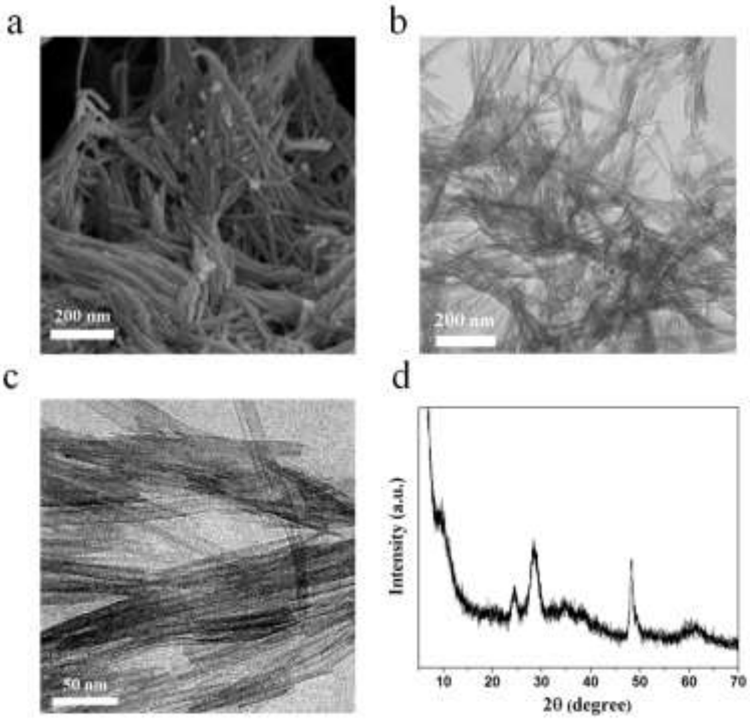

Supplementary Figure 9 | The morphology and phase characterization of layered titanate $Na_2Ti_3O_7$ nanotubes. (a) SEM image. (b-c) TEM images. (d) XRD pattern. $Na_2Ti_3O_7$ sample was synthesized according to a literature procedure[1]. The sample exhibited a typical morphological feature of thin nanotubes.

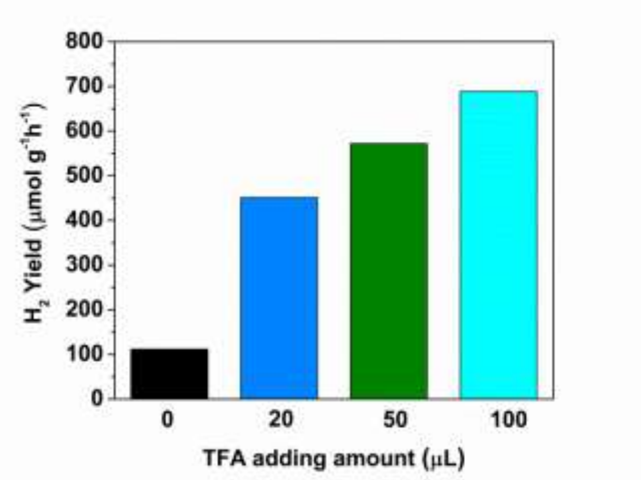

Supplementary Figure 10 | The comparison of photocatalytic H_2 evolution activity of layered titanate $Na_2Ti_3O_7$ nanotubes. Reaction condition: 50 mg catalyst, 200 mL 20 vol% methanol aqueous solution with different amounts of TFA.

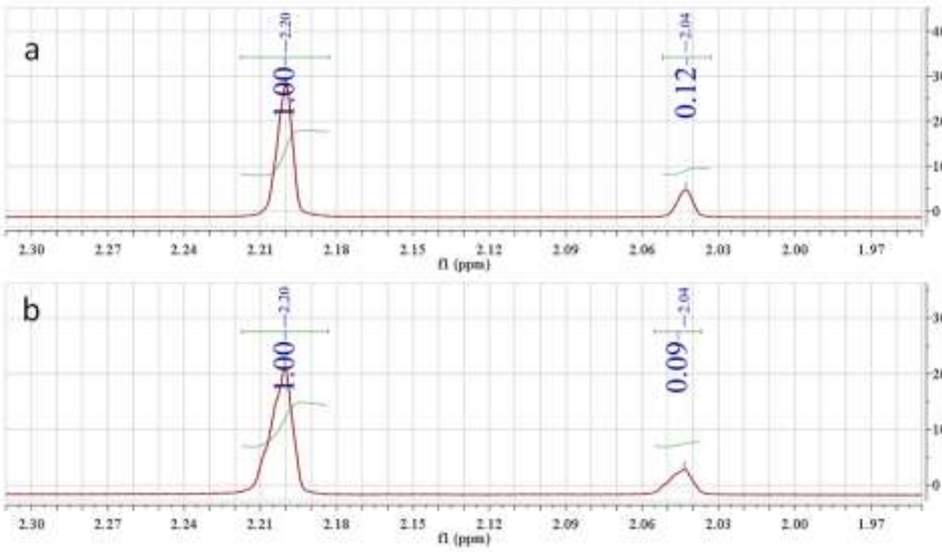

Supplementary Figure 11 | The comparison of ^{1}H NMR spectra (a) before and (b) after 12h photocatalytic experiment. Condition: 50 mg catalyst, 100 μL acetic acid in 200ml 20 vol % methanol aqueous solution. Before the measurement, a known amount of acetone (δ=2.20 ppm) was added as an internal standard.

Supplementary Table 1 | Molar ratio of adsorbed TFA/$K_4Nb_6O_{17}$ when adding different amount of TFA.

Amount of TFA/mL	0.05	0.1	0.12	0.15
Molar ratio of TFA/ $K_4Nb_6O_{17}$ /%	5.65	10.18	10.38	10.13

Supplementary Note 1

A dynamic equilibrium exists between the free and adsorbed TFA, and it is the adsorbed TFA that transfers the hole and thus plays the key role in the photocatalytic process. As illustrated in **Supplementary Table 1**, the molar ratio of adsorbed TFA/$K_4Nb_6O_{17}$ increased with the amount of TFA in low concentrations. When the amount of TFA exceeded 100 μL, the ratio underwent negligible change. And the maximum molar ratio is consistent with XPS result (26.03%, note: the result is relatively large due to the higher surface sensitivity of XPS technique in the depth range of 1~10 nm). Combined with the experimental results that no further improvement of the photocatalytic performance was observed when the amount of TFA exceeds 100 μL, it is safe to infer that TFA has reached the saturated adsorption on $K_4Nb_6O_{17}$ when 100 μL TFA is added.

Supplementary Note 2

To verify the reversibility of TFA, ^{19}F nuclear magnetic resonance spectroscopy (NMR) using NH_4BF_4 as an internal standard was applied to detect the fluoride species and quantitatively determine

the amount of TFA before and after the photocatalysis reaction. As illustrated in **Supplementary Fig. 2**, the NMR spectra before and after the photocatalysis reaction are consistent, both containing a single peak at δ= -75.6 ppm for CF_3COOH and a quartet centered at δ= -150.1 ppm for NH_4BF_4. No new fluoride species were observed after the reaction. Moreover, according to the quantitative results from the same NH_4BF_4 internal standard, the integral area ratio of TFA/NH_4BF_4 before and after the reaction was both 0.49, further illustrating that TFA was not consumed but reversible during the photocatalytic process.

Supplementary Note 3

As shown in **Supplementary Fig. 5**, ESR spectra during photocatalysis reaction of $K_4Nb_6O_{17}$ in DMPO aqueous solution showed the characteristic quartet of DMPO-HO• adduct. While before irradiation, no signals were observed, indicating that •OH radicals derived from the reaction of holes with OH^- or H_2O[2]. Since the observation of DMPO-HO• adduct, a typical spin trapping product by reaction of •OH radical with DMPO that has been reported by numerous literatures[3-5], it is reasonable to confirm the high stability of DMPO and rule out the formation of DMPO radical anion during the photocatalysis reaction. Thus, we exclude the possibility that DMPO-TFA adduct was formed by reaction of DMPO radical anion with TFA. Furthermore, when adding TFA to the above photocatalytic system after irradiation, we did not observe any new peaks in the ESR spectrum except relatively low signal intensity. The present observation further demonstrated that DMPO-TFA adduct formed by reaction of TFA radical anion with DMPO, rather than TFA with any DMPO derivatives.

Supplementary Note 4

As illustrated in **Supplementary Fig. 8**, for both KNbO and KNbO-TFA (where KNbO denotes $K_4Nb_6O_{17}$), the Pt loading accelerates the overall electron transfer (from the TA kinetics comparison: 71 ± 7 ps for Pt/KNbO vs. 126 ± 11 ps for KNbO; 108 ± 11 ps for Pt/KNbO-TFA vs. 312 ± 37 ps for KNbO-TFA) as well as the overall radiative electron–hole recombination (from the PL kinetics comparison: 1.69 ± 0.01 ns for Pt/KNbO vs. 3.0 ± 0.1 ns for KNbO; 3.18 ± 0.08 ns for Pt/KNbO-TFA vs. 8.2 ± 0.4 ns for KNbO-TFA). This is understandable because the Pt loading opens an additional electron-transfer channel (from the conduction band of KNbO to Pt) apart from the electron-trapping states of KNbO. As in the comparison of KNbO and KNbO-TFA, the comparison of Pt/KNbO and Pt/KNbO-TFA confirms again that the presence of TFA promotes the hole transfer process (from the lower hole-trapping states to the environment) as the situation of the lower hole-trapping states being effectively vacated via the TFA anion-accelerated hole transfer will eventually decelerate the electron–hole recombination process (cf. PL results) and hence decelerate the electron transfer process from the conduction band of KNbO to its electron-trapping states (cf. TA results). It is evident that, with or without the Pt loading, all the TA results are commensurate with and echo well to the transient PL measurements. The comparative analysis of these self-consistent results collected from our systematic PL and TA measurements is believed to well support the conclusion that TFA mainly influences the hole transfer process.

Supplementary Note 5

According to the literatures[6,7], both acetic acid and oxalic acid undergo irreversible oxidation. As reported by Armstrong, even 80% of the overall $CH_3COO•$ undergo rapid subsequent decomposition, and analogous reaction occurs with $C_2O_4•^{-}$.

$$CH_3COO^{-} + h^{+} \rightarrow CH_3COO• \rightarrow •CH_3 + CO_2 \quad (1)$$

$$C_2O_4^{2-} + h^{+} \rightarrow C_2O_4•^{-} \rightarrow COO•^{-} + CO_2 \quad (2)$$

The amount of acetic acid was quantitatively determined by ^1H NMR using acetone as the internal standard. As shown in **Supplementary Fig. 11**, ^1H NMR of the solution after 12h photocatalytic reaction exhibited a decreased integral area ratio of acetic acid/acetone, indicating the consumption of acetic acid in the photocatalytic process. The amount of oxalic acid was determined by precipitation titration with $CaCl_2$, and 37 % loss was observed after the photocatalysis reaction.

In contrast, for TFA, the extreme instability of $CF_3•$ brings great difficulties to the decomposition of $CF_3COO•$ in thermodynamics, and thus $CF_3COO•$ possesses a much better stability.

$$CF_3COO^{-} + h^{+} \rightarrow CF_3COO• \quad (3)$$

From the perspective of reversibility, neither acetic acid nor oxalic acid meets the requirements of molecular co-catalyst, even though both oxalic acid and acetic acid also cause over tenfold enhancement in the H_2 production rate.

Supplementary Methods

Characterization methods: The samples were characterized by X-ray powder diffraction (XRD) using a Philips X'Pert Pro Super diffractometer equipped with graphite-monochromatized Cu-Kα radiation (λ = 1.54178 Å). The scanning electron microscopy (SEM) images were performed on a Zeiss Supra 40. Transmission electron microscopy (TEM) images were taken on H-7650 (Hitachi, Japan) operated at an acceleration voltage of 100 kV. High-resolution transmission electron microscope (HRTEM) images were obtained on JEOL-2010 operating at an acceleration voltage of 200 kV. The UV–Vis absorption spectra were recorded on a Perkin Elmer Lambda 950 spectrophotometer. X-ray photoelectron spectroscopy (XPS) measurements were performed on a VG ESCALAB MK II X-ray photoelectron spectrometer with an excitation source of Mg Kα = 1253.6 eV. Mott–Schottky plot was measured in degassed 0.5 M Na_2SO_4 solution (pH = 6.6) at a frequency of 10 Hz. The applied potential ranges from –0.5 to +0.5 V.

Ultrafast transient absorption (TA) spectroscopy characterizations: The femtosecond pump–probe experiments were performed on a modified ExciPro system (CDP) in combination with an ultrafast amplified laser system (Coherent). All the measurements were performed under ambient conditions. The pump pulses (centre wavelength at 300 nm for this study; pulse energy ~8 μJ at the sample cell) were delivered by an optical parametric amplifier (TOPAS-800-fs), which was excited by a Ti:sapphire regenerative amplifier (Legend Elite-1K-HE; centre wavelength 800 nm, pulse duration 25 fs, pulse energy 3 mJ) seeded with a mode-locked Ti:sapphire laser system (Micra 5) and pumped with a 1-kHz Nd:YLF laser (Evolution 30). The probe pulses (<1 μJ/pulse at the sample cell) were provided by a stable white-light continuum (WLC) (390−610 nm for this study) that was generated by focusing the 800-nm beam (split from the regenerative amplifier by a portion of 10%) in a rotating CaF_2 crystal. The linear chirp of the WLC spectrum was compensated with the aid of the cross-phase modulation signals recorded on a liquid hexane sample using an ExiPro 2.6 software. The WLC pulse was free of pre-pulsing and after-pulsing, as verified by our routine pulse characterizations. The pump and probe

beams were collinearly polarized. The instrument response function was determined to be ~100 fs by cross-correlating the pump and probe pulses at the sample cell. Precise spatial overlap of the pump and probe beams (diameters ~800 and 300 μm, respectively) at the centre of the 1.2-mm-thick sample cell (quartz) was attained by optimizing the transient signals with the aid of a laser beam analyzer (BG-USB-SP620, Ophir-Spiricon). The delay times between the pump and probe pulses were varied by a motorized optical delay line (minimum step 1.56 fs; maximum delay ~2 ns). The delay time zero was determined by cross-correlating the pump and probe pulses at the sample cell in situ and also carefully cross-checked with other chemical samples such as the DCM and LDS698 dyes. A chopper operating at 500 Hz was used to modulate the pump pulses such that the transient spectra with and without the pump pulses can be recorded alternately. The WLC probe beam was first split into two tiny portions to synchronize the chopper and monitor the stability of the probe pulses using two separate photodiode detectors, and then separated into two parts (intensity ratio ~70:30), with the 70% part focused on the sample cell and overlapped with the pump beam yielding a transmitted probe signal, while the 30% part focused onto another place of the sample cell to serve as a reference signal for achieving a best signal-to-noise ratio. The samples under investigation were the $K_4Nb_6O_{17}$ film immersed in a methanol/water (1:4 vol%) mixed solution and the $K_4Nb_6O_{17}$ film immersed in a methanol/water (1:4 vol%) mixed solution with a tiny amount of TFA addition (0.5 mL/L). The sample cell was mounted on a rotating stage to ensure that the photoexcited volume of the sample was kept fresh during the course of the measurements. The transient absorption signals (i.e., absorbance changes, or ΔAbs. in short) were visualized by a 1024-pixel imaging spectrometer (CDP2022i) and further processed by the ExiPro 2.6 software.

Photoluminescence (PL) spectroscopy characterizations: The photoluminescence (PL) emission spectra were obtained on a FLUOROLOG-3-TAU fluorescence spectrometer (Horiba) upon excitation at 315 nm. The ns-domain time-resolved PL spectra (excited at 315 nm and monitored at the 430-nm emission) were recorded on an FLS920 fluorescence spectrometer (Edinburgh).

Electron spin resonance (ESR) characterizations: The spin trapping experiments were performed in deoxygenated solutions using a JES-FA200 electron spin resonance (ESR) spectrometer at room temperature. DMPO was used as a spin trapping agent. The irradiation experiments were carried out with a Xe lamp (500 W, USHIO Optical Modulex SX-U1501XQ).

Calculation details of the redox potential E (TFA•/TFA⁻):

The calculations were carried out with the CBS-QB3 method, which is a composite method starting from a geometry optimization at B3LYP/6-311G(2d,d,p) followed by a series of high level single-point energy corrections including the complete basis set extrapolation[8,9]. Solvent effects were evaluated by using the Polarizable Continuum Model (PCM) [10-12] with water as the solvent. We adopted the solvent accessible surface (SAS) models of molecular surface representing the solute–solvent boundary. All calculations were carried out with the Gaussian 09 program package [13].

$$\Delta G(\text{IE, sol}) = G(\text{radical, sol}) - G(\text{anion, sol}) = 6.19 \text{ eV}$$

Since ΔG for the SHE is 4.28 eV[14],

$$\Delta G(\text{TFA}^- \rightarrow \text{TFA}\bullet) = \Delta G(\text{IE, sol}) - \Delta G_{SHE} = 6.19 - 4.28 = 1.91 \text{ eV},$$

and thus the redox potential E(TFA•/TFA^-) is 1.91 V.

Supplementary References

1. Morgan, D. L., Zhu, H.-Y., Frost, R. L. & Waclawik, E. R. Determination of a Morphological Phase Diagram of Titania/Titanate Nanostructures from Alkaline Hydrothermal Treatment of Degussa P25. *Chem. Mater.* **20**, 3800-3802 (2008).
2. Yu, J., Qi, L. & Jaroniec, M. Hydrogen Production by Photocatalytic Water Splitting over Pt/TiO_2 Nanosheets with Exposed (001) Facets. *J. Phys. Chem. C* **114**, 13118-13125 (2010).
3. Finkelstein, E., Rosen, G. M. & Rauckman, E. J. Spin trapping. Kinetics of the reaction of superoxide and hydroxyl radicals with nitrones. *J. Am. Chem. Soc.* **102**, 4994-4999 (1980).
4. Shi, W., Zhang, X., He, S. & Huang, Y. CoFe2O4 magnetic nanoparticles as a peroxidase mimic mediated chemiluminescence for hydrogen peroxide and glucose. *Chem. Commun.* **47**, 10785-10787 (2011).
5. Fang, G., Gao, J., Dionysiou, D. D., Liu, C. & Zhou, D. Activation of Persulfate by Quinones: Free Radical Reactions and Implication for the Degradation of PCBs. *Environ. Sci. Technol.* **47**, 4605-4611 (2013).
6. Kraeutler, B. & Bard, A. J. Heterogeneous photocatalytic decomposition of saturated carboxylic acids on titanium dioxide powder. Decarboxylative route to alkanes. *J. Am. Chem. Soc.* **100**, 5985-5992 (1978).
7. Yu, D., Rauk, A. & Armstrong, D. A. Radicals and ions of formic and acetic acids: an ab initio study of the structures and gas and solution phase thermochemistry. *J. Chem. Soc., Perkin Trans.*, 2207-2215 (1994).
8. Montgomery, J. A., Frisch, M. J., Ochterski, J. W. & Petersson, G. A. A complete basis set model chemistry. VI. Use of density functional geometries and frequencies. *J. Chem. Phys.* **110**, 2822−2827 (1999).
9. Montgomery, J. A., Frisch, M. J., Ochterski, J. W. & Petersson, G. A. A complete basis set model chemistry. VII. Use of the minimum population localization method. *J. Chem. Phys.* **112**, 6532−6542 (2000).
10. Cancès, E., Mennucci, B. & Tomasi, J. A new integral equation formalism for the polarizable continuum model: Theoretical background and applications to isotropic and anisotropic dielectrics. *J. Chem. Phys.* **107**, 3032-3041 (1997).
11. Cossi, M., Barone, V., Mennucci, B. & Tomasi, J. Ab initio study of ionic solutions by a polarizable continuum dielectric model. *Chem. Phys. Lett.* **286**, 253−260 (1998).
12. Mennucci, B. & Tomasi, J. Continuum solvation models: A new approach to the problem of solute's charge distribution and cavity boundaries. *J. Chem. Phys.* **106**, 5151−5158 (1997).
13. M.J. Frisch, *et al.*, Gaussian 09, Revision B.01, Gaussian Inc., Wallingford CT, 2009.
14. Isse, A. A. & Gennaro, A. Absolute potential of the standard hydrogen electrode and the problem of interconversion of potentials in different solvents. *J. Phys. Chem. B* **114**, 7894−7899 (2010).
